# Immune Infiltration of MMP14 in Pan Cancer and Its Prognostic Effect on Tumors

**DOI:** 10.3389/fonc.2021.717606

**Published:** 2021-09-17

**Authors:** Minde Li, Shaoyang Li, Lin Zhou, Le Yang, Xiao Wu, Bin Tang, Shenhao Xie, Linchun Fang, Suyue Zheng, Tao Hong

**Affiliations:** Department of Neurosurgery, The First Affiliated Hospital of Nanchang University, Nanchang, China

**Keywords:** MMP14, pan-cancer analysis, immune infiltration, prognosis, tumor immune microenvironment

## Abstract

**Background:**

Matrix metalloproteinase 14 (MMP14) is a member of the MMP family, which interacts with tissue inhibitors of metalloproteinase (TIMPs), and is involved in normal physiological functions such as cell migration, invasion, metastasis, angiogenesis, and proliferation, as well as tumor genesis and progression. However, there has been a lack of relevant reports on the effect of MMP14 across cancers. This study aims to explore the correlation between MMP14 and pan-cancer prognosis, immune infiltration, and the effects of pan-cancer gene mismatch repair (MMR), microsatellite instability (MSI), tumor mutational burden (TMB), DNA methylation, and immune checkpoint genes.

**Methods:**

In this study, we used bioinformatics to analyze data from multiple databases, including The Cancer Genome Atlas (TCGA), ONCOMINE, and Kaplan–Meier plotter. We investigated the relationship between the expression of MMP14 in tumors and tumor prognosis, the relationship between MMP14 expression and tumor cell immune infiltration, and the relationship between MMR gene MMR, MSI, TMB, DNA methylation, and immune checkpoint genes.

**Results:**

MMP14 expression is highly associated with the prognosis of a variety of cancers and tumor immune invasion and has important effects on pan oncologic MMR, MSI, TMB, DNA methylation, and immune checkpoint genes.

**Conclusion:**

MMP14 is highly correlated with tumor prognosis and immune invasion and affects the occurrence and progression of many tumors. All of these results fully indicate that MMP14 may be a biomarker for the prognosis, diagnosis, and treatment of many tumors and provide new ideas and direction for subsequent tumor immune research and treatment strategies.

## Introduction

Matrix metalloproteinase 14 (MMP14), also called membrane type 1 metalloproteinase (MT1-MMP), is a member of the MT-MMP subfamily. MMPs are a class of zinc-binding proteinases that contribute to tumor metastasis by degrading the extracellular matrix (ECM) (1). As a member of the first MMP family to be identified, MMP14 is involved in many biological processes in cells, including proliferation, invasion, vascular production, and basement membrane remodeling (2, 3). In general, the vast majority of MMPs are tissue-deficient and have no proenzyme activity, requiring further activation to produce biological activity, but MMP14 is an exception, as it does not require additional activation and can be directly present in its active form on the cell membrane (4). It has been reported that the increased expression and activity of MMP14 in tumor cells are directly related to their enhanced cell migration ability (5). It has also been reported that as a tumor promoter, MMP14 acts by inhibiting cell adhesion molecules, and tumor necrosis factor-α (TNF-α) is one of its acting factors (6). Studies have shown that MMP14 is involved in the progression of cervical cancer (CC) by promoting angiogenesis, invasion, and lymph node metastasis, and it has also been reported that MMP14 overexpression is associated with poor prognosis of cervical cancer (7–9). At the same time, a large number of studies have shown that MMP14 is closely related to the invasion, migration, and angiogenesis of ovarian cancer (10), gastric cancer (11), glioma (12), pancreatic cancer (12), hepatocellular carcinoma (13), colon cancer (14), and melanoma (15). However, there has been no study on the effect of MMP14 on the immune infiltration and prognosis of pan tumor cells, which needs to be addressed.

The relationship between tumors and the immune system is complex, and the mechanism of interaction is currently unknown (16). The tumor microenvironment plays an important role in modulating tumors and the immune system, a large proportion of which are immune-infiltrating cells (17). It has long been widely believed that the immune system plays a significant positive role in antitumor activities (16), but now a dissenting view has emerged that the immune system can help tumor cells escape predation and that this effect is attributed to the tumor microenvironment (18–20). In recent years, with increased knowledge of immunity, there have been many studies investigating the value of immune-infiltrating cells in tumors (21, 22). Factors including cytotoxic T lymphocyte-associated antigen 4 (CTLA4), programmed death-1 (PD-1), and programmed death-ligand 1 (PD-L1) have been shown to play important roles in tumor treatment (19, 23). However, the proportion of tumors that responds to these immunonode inhibitors is reported to be very small (19). Therefore, it is very important and urgent to strengthen research on the treatment of the immune infiltration microenvironment.

In this study, we used bioinformatics to analyze data from multiple databases, including The Cancer Genome Atlas (TCGA), ONCOMINE, and Kaplan–Meier (KM) plotter. We investigated the relationship between the expression of MMP14 in tumors and tumor prognosis, the relationship between MMP14 expression and tumor cell immune infiltration, and the relationship between mismatch repair (MMR) gene MMR, microsatellite instability (MSI), tumor mutational burden (TMB), DNA methylation, and immune checkpoint genes.

## Materials and Methods

### MMP14 Expression in Human Cancers in ONCOMINE

In the ONCOMINE Database (www.oncomine.org), the p-value was set to 0.001, and the fold change was set to 2. Then, the mRNA expression levels of MMP12 in different types of tumors and their adjacent tissues were compared and analyzed.

### Prognostic Analysis of MMP14 in the Kaplan–Meier Plotter Database

KM plotter (https://kmplot.com/analysis/) is a visual graphics platform facilitating analysis of 21 kinds of cancer, including breast cancer (BRCA) (n = 7,830), ovarian cancer (n = 2,190), lung cancer (n = 3,452), and gastric cancer (n = 1,440). We obtained clinical information on pan-cancer from TCGA database (24–26). Then, KM analysis was used to analyze the correlation between overall survival (OS), disease-specific survival (DSS), progression-free interval (PFI), disease-free interval (DFI) (24), and MMP14 expression in pan-cancer patients.

### Analysis of Immune Infiltration of MMP14 in the TIMER Database

TIMER (https://cistrome.shinyapps.io/timer/) is a very powerful and practical platform that can be easily applied to tumor immunity research and has the ability to visualize immune and genomics data (27). The data on the platform to date include more than 10,000 samples from 32 cancers in TCGA, with a large number of tumor-infiltrating immune cells (28). In this study, we evaluated the relationship between MMP14 expression and the level of immune infiltrate in 32 cancer types (six subsets: B cells, CD4+ T cells, CD8+ T cells, macrophages, neutrophils, and dendritic cells) and tumor purity.

### Correlation Analysis of MMP14 Expression With Mismatch Repair Mutations and DNA Methylation

DNA MMR can affect tumor genesis by correcting DNA replication errors and reducing chromosomal rearrangement (29, 30). MLH1 (MutL Protein Homolog 1), MSH2 (MutS protein homologue 2), MSH6 (MutS homologue 6), EPCAM (Epithelial cell adhesion molecular), and PMS2 (PMS1 homologue 2) are the five important genes of MMR. We obtained MMR-related tumor data from TCGA database, including these five important genes and MMP14. The expression levels of the MMR gene and MMP14 were analyzed by the Spearman correlation method. In addition, DNA methylation is an important factor affecting genes. In this study, the Spearman correlation method was used to analyze the relationship between the expression levels of DNMT1 (DNA methyl transferase 1), DNMT2, DNMT3A, and DNMT3B and the expression of MMP14.

### Correlation Analysis of MMP14 Expression With Tumor Mutational Burden and Microsatellite Instability

TMB is defined as the total amount of DNA mutations produced by tumor cells (31–34). In this study, we extracted somatic cell data (MAF data) from TCGA database and used the “MAF Tools” R package for analysis. Through correlation processing, the total number of exon mutations was obtained; that is, the TMB of the tumor was determined. MSI is defined as a phenomenon in which nucleotides of repeating DNA fragments are added or lost (35, 36). The MSI score can be obtained from TCGA database. Then, Spearman analysis was used to analyze the correlation between MMP14 expression and TMB or MSI.

### Statistical Analysis

In the analysis of differential expression of MMP14 in tumors and normal tissues, we used the ONCOMINE database to analyze p-values, fold changes, and gene ranks. Meanwhile, in the survival analysis, the KM method was used to analyze the prognosis of patients according to univariate Cox regression analysis and different expression levels of MMP14. Spearman correlation analysis was also used to evaluate the correlation between MMP14 expression and methyltransferase levels, MMR gene expression levels, and immune checkpoint gene expression. R > 0.20 was positively correlated, and p < 0.05 was considered statistically significant.

## Results

### Differential Analysis of MMP14 Expression in Pan-Cancer Tissues and Normal Tissues

**Figure 1:**
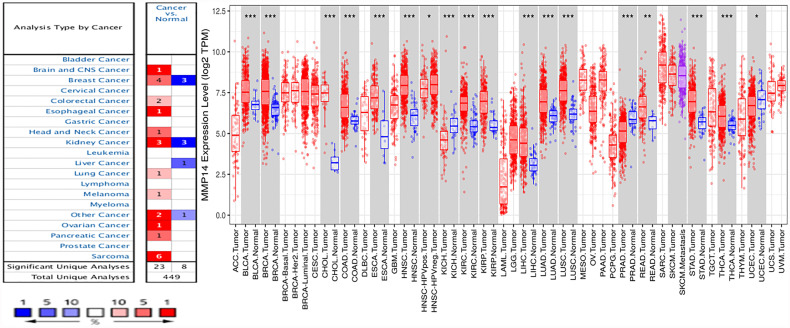
Differences in MMP14 expression in tumor tissues and normal tissues across cancers. **(A)** The Oncomine database was used to analyze the expression of MMP14 in tumor tissues and normal tissues. The number in each cell is the total amount in the dataset. **(B)** Analysis and comparison of MMP14 expression in different tumor tissues and normal tissues in TCGA database. *P < 0.05, **P < 0.01 ***P < 0.001.

To analyze the expression levels of MMP14 mRNA in normal tissues and tumors, we analyzed relevant data from the ONCOMINE and TIMER databases. In the ONCOMINE database, compared to normal tissue, the results showed that the expression of MMP14 was higher in the brain and central nervous system (CNS) cancer, BRCA, colorectal carcinoma, esophageal cancer, head and neck cancer, kidney cancer, lung cancer, melanoma, ovarian cancer, pancreatic cancer, and sarcoma (SARC) (**Figure 1A**). MMP14 expression was decreased only in liver cancer.

At the same time, we further confirmed the expression of MMP14 in various cancers compared to normal tissues using the TIMER database (**Figure 1B**). The results showed that bladder cancer (BLCA), BRCA, cholangiocarcinoma (CHOL), colon adenocarcinoma (COAD), esophageal carcinoma (ESCA), kidney renal clear cell carcinoma (KIRC), kidney renal papillary cell carcinoma (KIRP), liver hepatocellular carcinoma (LIHC), lung adenocarcinoma (LUAD), lung squamous cell carcinoma (LUSC), rectum adenocarcinoma (READ), stomach adenocarcinoma (STAD), and thyroid carcinoma (THCA) tissues were significantly increased compared to normal tissues. Tumors with reduced MMP14 expression compared to normal tissue included head and neck squamous cell carcinoma (HNSC), kidney chromophobe (KICH), prostate adenocarcinoma (PRAD), and uterine corpus endometrial carcinoma (UCEC).

### Prognostic Role of MMP14 in Different Tumors

**Figure 2:**
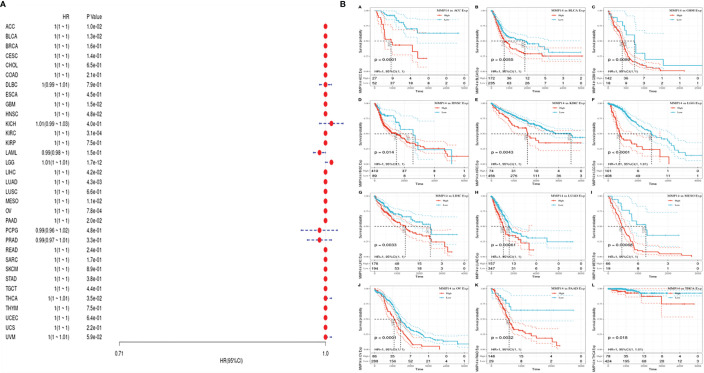
The relationship between the expression of matrix metalloproteinase 14 (MMP14) and the overall survival (OS) and prognosis of pan-cancer was analyzed. **(A)** The correlation between MMP14 expression and OS in different cancer types of The Cancer Genome Atlas (TCGA) was analyzed. The red part represents the risk ratio. Due to the limited sample size, the parameters and hazard ratio could not be calculated with short bars, and the red font indicates p < 0.05. **(B)** Kaplan–Meier analysis was used to generate a survival curve for the prognostic effect of MMP14 expression on pan-cancer. OS, overall survival.

To investigate the prognostic effect of MMP14 in different tumors, we analyzed data from different databases. First, the OS, DSS, disease-free survival (DFS), and DFI of MMP14 were analyzed by a univariate Cox proportional risk regression model in TCGA database. KM plotter survival curves were accordingly plotted for tumors with significant prognosis (**Figure 2**).

First, we used gene expression profile data to analyze the relationship between MMP14 expression and tumor prognosis in 33 tumors in TCGA database. Univariate survival analysis resulted in a forest plot of prognosis for 33 tumors, as shown in **Figure 2A**. KM plot 2b was also plotted with a significant correlation with MMP14 expression. Low MMP14 expression was observed in adrenocortical carcinoma (ACC), BLCA, glioblastoma multiforme (GBM), HNSC, KIRC, brain lower grade glioma (LGG), LIHC, LUAD, mesothelioma (MESO), ovarian serous cystadenocarcinoma (OV), and pancreatic adenocarcinoma (PAAD), and OS in THCA was positively related to prognosis.

**Figure 3:**
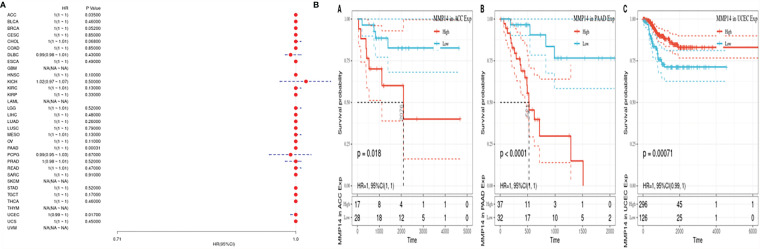
The expression of matrix metalloproteinase 14 (MMP14) was correlated with disease-free survival (DFS), and prognosis was analyzed by disease-free interval (DFI). The expression of MMP14 and the overall survival rate of pan-cancer were analyzed by DFI. **(A)** Correlation analysis of MMP14 expression and DFI in different cancer types of The Cancer Genome Atlas (TCGA). The red part represents the risk ratio. Due to the limited sample size, the parameters and hazard ratio could not be calculated with short bars, and the red font indicates p < 0.05. **(B)** Kaplan–Meier analysis was used to generate a survival curve for the prognostic effect of MMP14 expression on pan-cancer. DFI, disease-free interval survival.

Given the statistical data for tumor death, we performed TCGA database analysis of 33 kinds of tumor DSS, constructed a forest plot, and observed a significant relationship with KM, as shown in **Figure 3**. From **Figure 3**, we can identify factors other than tumor death, including that lower expression of MMP14 was positively related to the prognosis of tumor types such as ACC, PAAD, and UCEC.

**Figure 4:**
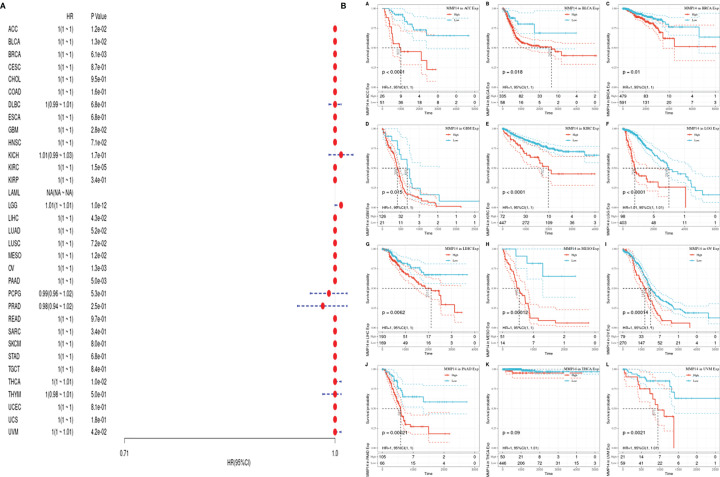
The expression of matrix metalloproteinase 14 (MMP14) and the prognosis of disease-specific survival (DSS) were analyzed. The relationship between MMP14 expression and disease-free survival was analyzed by DSS. The expression of MMP14 and the overall survival rate of pan-cancer were analyzed by DSS. **(A)** Correlation analysis of MMP14 expression and DSS in different cancer types of The Cancer Genome Atlas (TCGA). The red part represents the risk ratio. Due to the limited sample size, the parameters and hazard ratio could not be calculated with short bars, and the red font indicates p < 0.05. **(B)** Kaplan–Meier analysis was used to generate a survival curve for the prognostic effect of MMP14 expression on pan-cancer. DSS, disease-specific survival.

Considering the influence of disease factors on survival analysis results, DFS, which is applied to evaluate radical surgery, is generally used to indicate the time from treatment to recurrence. We performed DFS analysis on the above data and obtained a forest plot and KM map with significant prognosis, as shown in **Figure 4**. After radical surgery, the lower expression of MMP14 had significant effects on the prognosis of tumors, including ACC, BLCA, BRCA, GBM, KIRC, LGG, LIHC, MESO, OV, PAAD, THCA (Thyroid carcinoma), and uveal melanoma (UVM).

**Figure 5:**
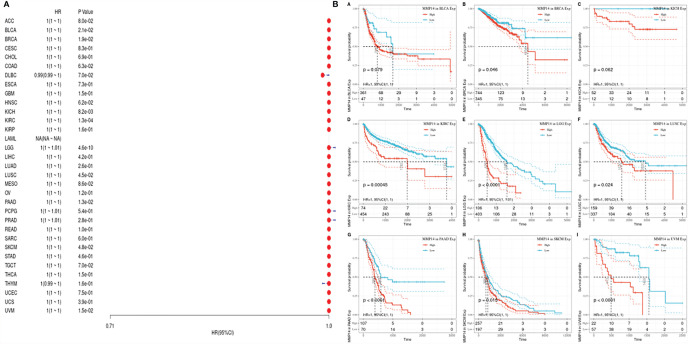
The relationship between MMP14 expression and PFI prognosis was analyzed. The expression of MMP14 and prognosis of pan cancer disease-specific survival were analyzed by PFI. The relationship between MMP14 expression and disease-free survival was analyzed by DSS. The expression of MMP14 and the overall survival rate of pan cancer patients were analyzed by PFI. **(A)** Correlation analysis of MMP14 expression and PFI in different cancer types of TCGA; the red part represents the risk ratio. Due to the limited sample size, the parameters and hazard ratio could not be calculated with short bars, and the red font indicates P < 0.05. **(B)** Kaplan–Meier analysis was used to generate a survival curve for the prognostic effect of MMP14 expression on pan cancer. PFI, progression-free interval survival.

PFS is an indicator of how well cancer responds after palliative care. We analyzed the PFS, and the results are shown (**Figure 5**). From **Figure 5**, we see that after palliative therapy, the lower expression of MMP14 obviously affected the prognosis of BLCA, BRCA, KICH, KIRC, LGG, and LUSC.

### Relationship Between the Expression of MMP14 and Immune Infiltration in Diffuse Tumors

**Figure 6:**
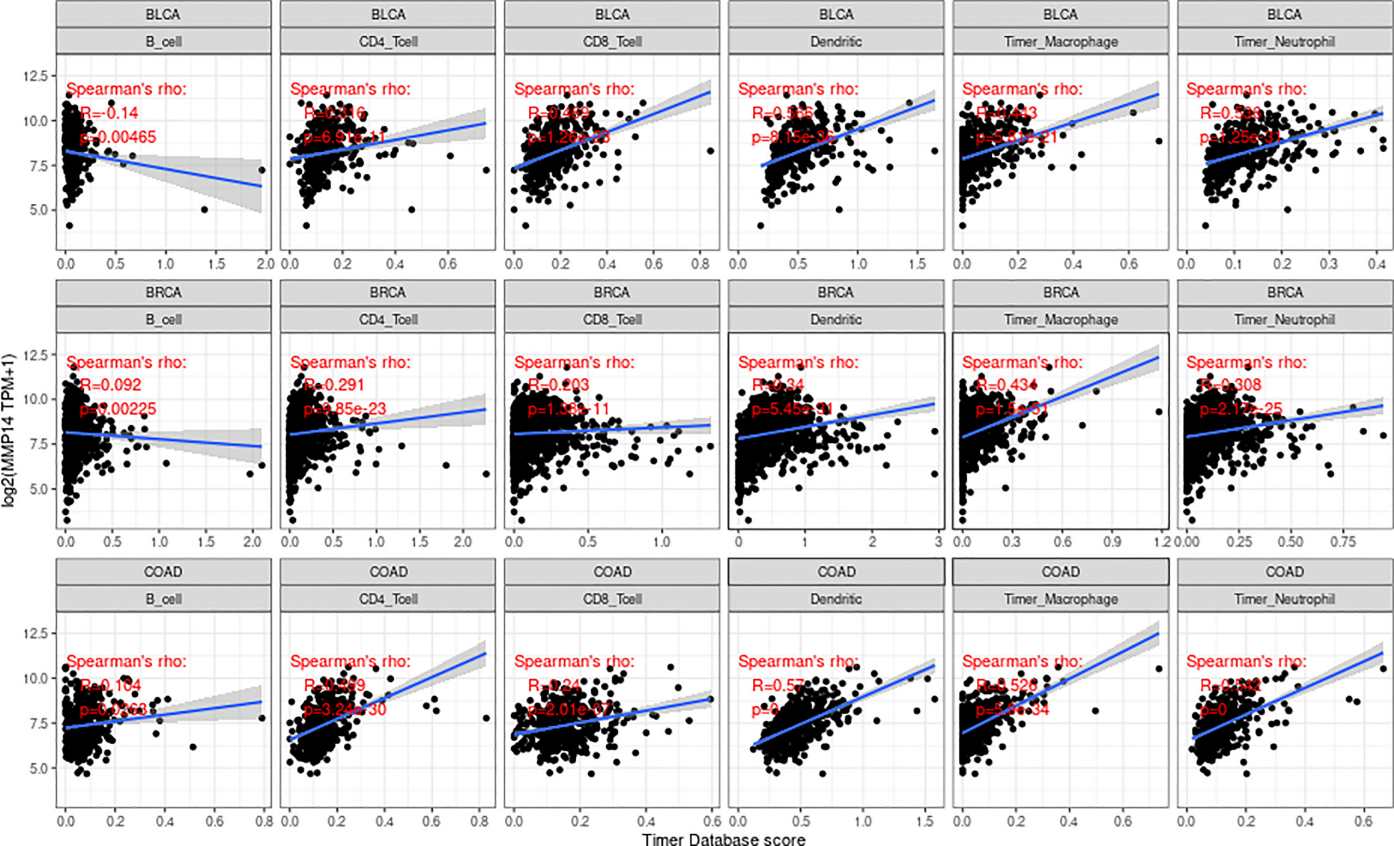
The expression of matrix metalloproteinase 14 (MMP14) in bladder urothelial carcinoma (BLCA), breast invasive carcinoma (BRCA), and colon adenocarcinoma (COAD) was correlated with the level of immune infiltration (B cells, CD4 T cells, CD8 T cells, macrophages, dendritic cells, and neutrophils). Only in BLCA was the expression of MMP14 negatively correlated with B-cell infiltration, and the others were positively correlated with the six kinds of immune cell infiltration.

We obtained score data for six infiltrating immune cells in 33 tumors from the TIMER database. Then, the expression of MMP14 was correlated with six species (B cells, CD4 T cells, CD8 T cells, macrophages, dendritic cells, and neutrophils), and the correlation between the scores of immune cells was analyzed (**Supplementary Figures S1–S4**). We found that the expression of MMP14 was most significantly associated with the infiltration of immune cells in three tumors: BLCA, BRCA, and COAD (**Figure 6**).

**Figure 7:**
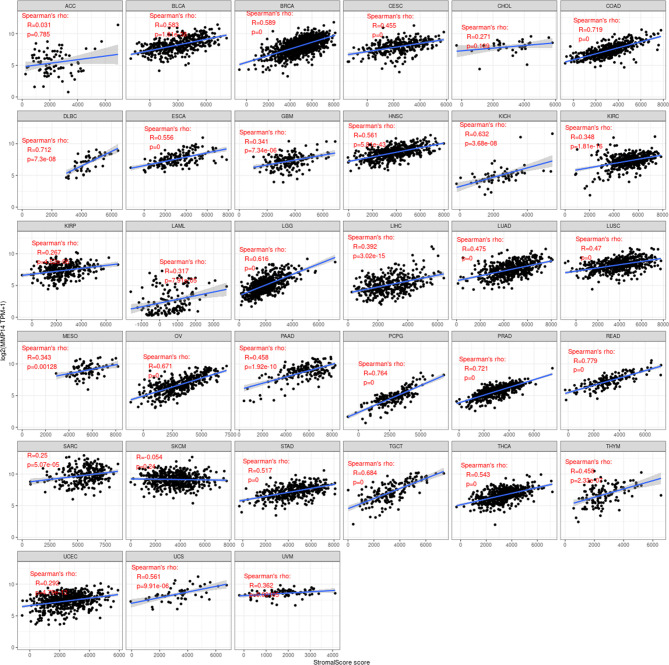
Relationship between the expression of matrix metalloproteinase 14 (MMP14) and immune score in pan-cancer. Only in adrenocortical carcinoma (ACC), skin cutaneous melanoma (SKCM), testicular germ cell tumor (TGCT), and thymoma (THYM) was the expression of MMP14 negatively correlated with immune cell infiltration, and the others were positively correlated with immune cell infiltration (in the case of p < 0.05).

**Figure 8:**
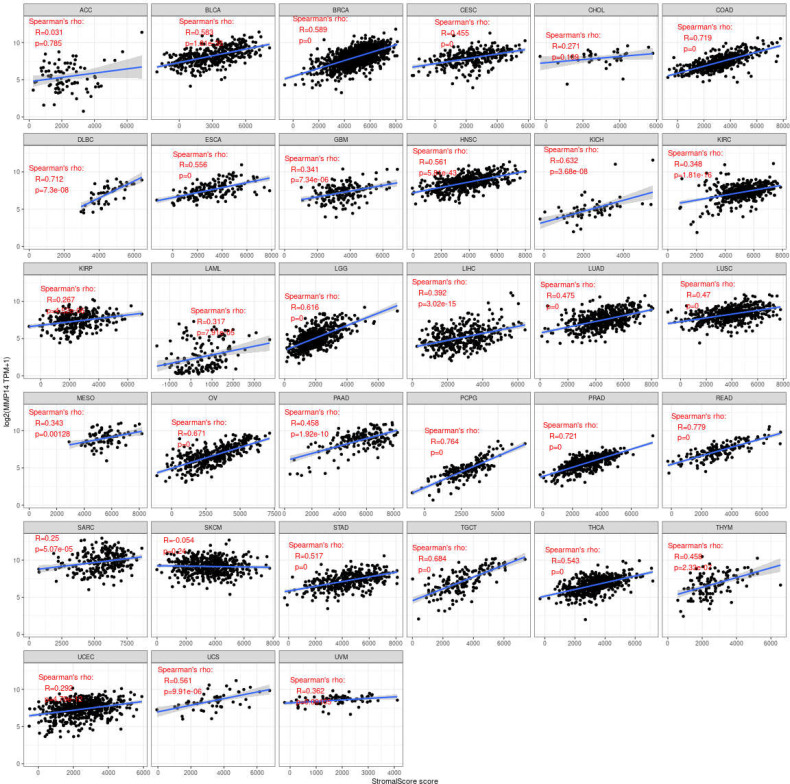
Relationship between the expression of matrix metalloproteinase 14 (MMP14) and stromal score in pan-cancer. Only in skin cutaneous melanoma (SKCM) was the expression of MMP14 negatively correlated with immune cell infiltration, and the others were positively correlated with immune cell infiltration (p < 0.05).

**Figure 9:**
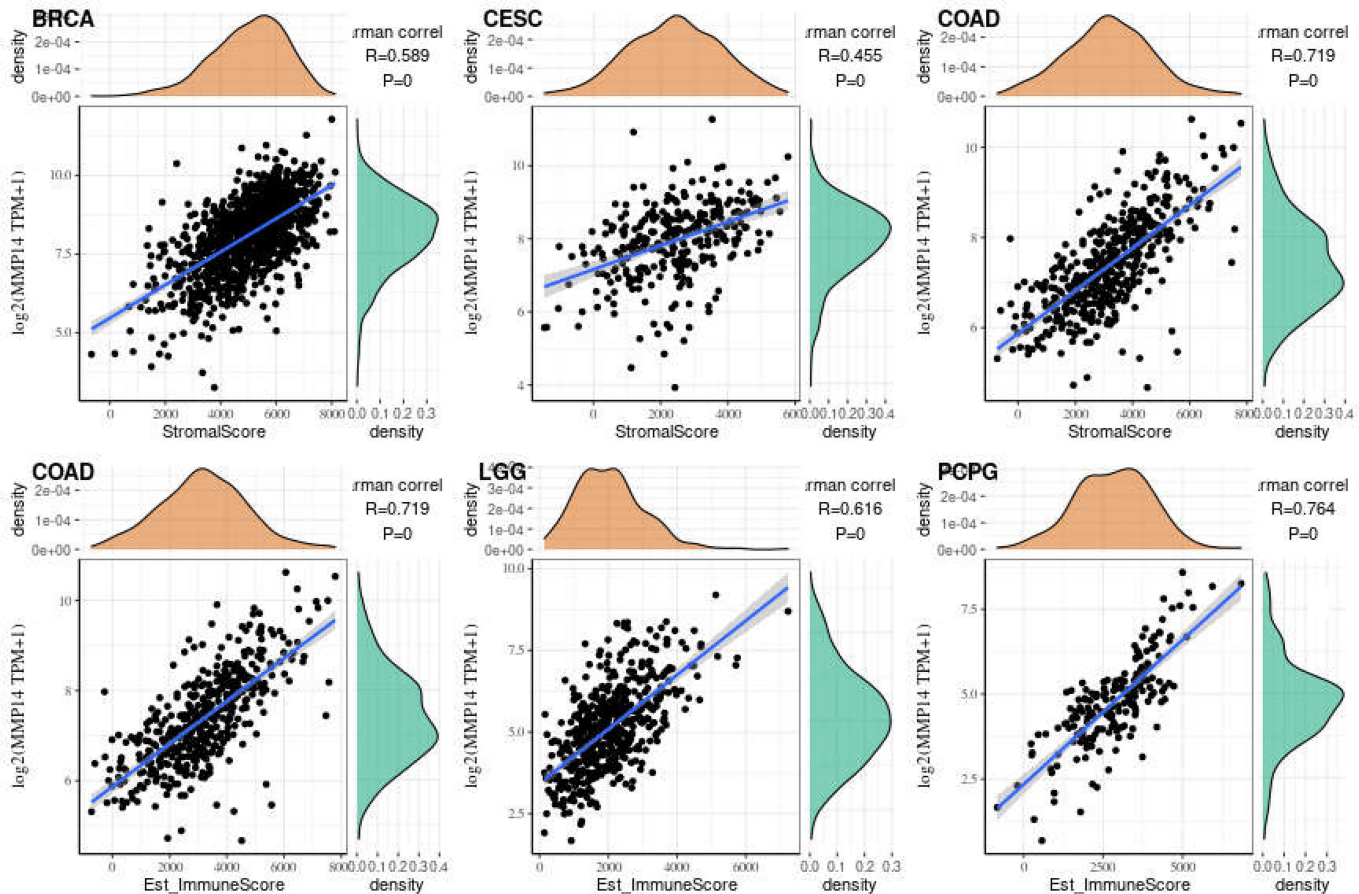
The three tumors with the highest correlation between matrix metalloproteinase 14 (MMP14) expression and immune score and stromal score in the pan-cancer analysis. The expression of MMP14 is positively correlated with the infiltration levels of these immune cells.

Immune cells and stromal cells are the two main cell types in the tumor microenvironment, and we analyzed them using the R package Estimate to determine each tumor sample immune score and score matrix. It was concluded that the MMP14 expression of 33 tumors and the immune score (ImmuneScore) relationship showed a significant correlation (p < 0.05), as the expression of MMP14 and the immune score were positively related in BLCA, BRCA, CESC (cervical cancer), COAD, lymphoid neoplasm diffuse large B-cell lymphoma (DLBC), ESCA, GBM, HNSC, KICH, KIRP, LGG, LIHC, LUAD, LUSC, OV, PAAD, pheochromocytoma and paraganglioma (PCPG), PRAD, READ, STAD, and THCA. At the same time, ACC, skin cutaneous melanoma (SKCM), testicular germ cell tumor (TGCT), and thymoma (THYM) were negatively correlated (**Figure 7**). In the relationship between the expression of MMP14 and StromalScore, the expression of MMP14 was positively and significantly correlated with the expression of the immune matrix (p < 0.05) for BLCA, BRCA, CESC, COAD, DLBC, ESCA, GBM, HNSC, KICH, KIRC, KIRP, acute myeloid leukemia (LAML), LGG, LIHC, LUAD, LUSC, MESO, OV, PAAD, PCPG, prostate adenocarcinoma (PARD), READ, SARC, STAD, testicular germ cell tumor (TGCT), THCA, THYM, uterine corpus endometrial carcinoma (USEC), uterine carcinosarcoma (UCS), and UVM. A negative correlation was found in SKCM (p = 0.24) (**Figure 8**). The three tumors with the most significant correlations between MMP14 expression and immune cell score (StromalScore) were BRCA, CESC, and COAD (**Figure 9A**), and those with ImmuneScore were COAD, LGG, and PCPG (**Figure 9B**). In conclusion, the expression of MMP14 is associated with immune infiltration.

### The Expression of MMP14 Is Associated With Immune Checkpoint Genes in Endemic Cancer

**Figure 10:**
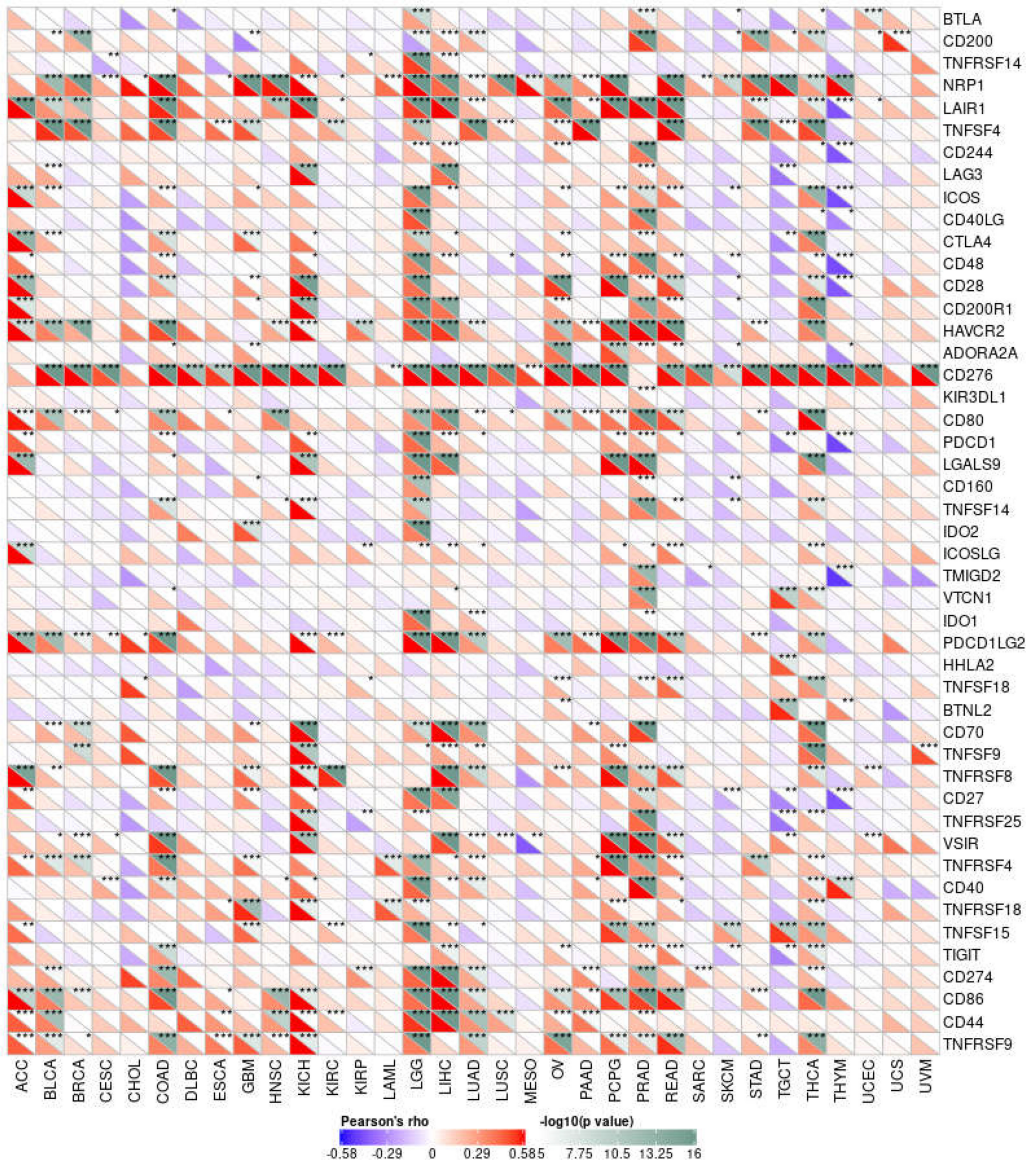
Correlation analysis of MMP14 expression and 47 immune checkpoint genes in endemic cancer.MMP14 was associated with 38 immune checkpoint genes in PRAD, 36 in LGG, 33 in THCA and 24 in KICH. We also found that the immune checkpoint gene CD276 was significantly associated with MMP14 expression in 28 of 33 tumors (*P < 0.05, **P < 0.01 ***P < 0.001).

From the above results, it can be seen that the expression of MMP14 is correlated with the level of immune infiltration, so we further studied the relationship between MMP14 and 47 common immune checkpoint genes (**Figure 10**). The results showed that the expression of MMP14 was associated with 41 immune checkpoint genes in PRAD, 36 in LGG, 33 in THCA, and 29 in KICH. We also found that the immune checkpoint gene CD276 was significantly associated with MMP14 expression in 28 of 33 tumors. The expression of MMP14 is associated with immune checkpoint genes in endemic cancer, and these results provide further evidence that MMP14 expression is associated with the level of immune invasion.

### The Expression of MMP14 Is Associated With Gene Mismatch Repair and DNA Methylation Levels in Endemic Cancer

**Figure 11:**
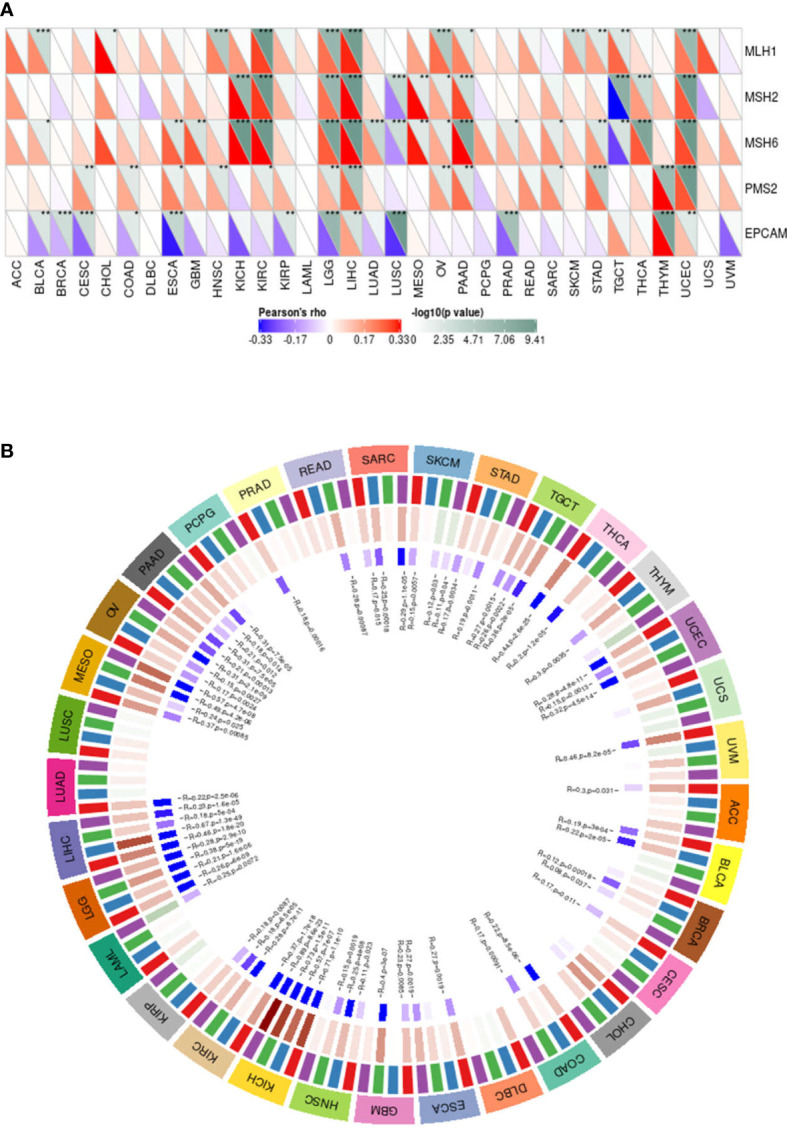
The expression of matrix metalloproteinase 14 (MMP14) was correlated with the expression of five mismatch repair (MMR) genes and four DNA methyltransferases. **(A)** Spearman correlation analysis was used to analyze the correlation between MMR gene expression and MMP14 expression in tumors (*p < 0.05, **p < 0.01, **p < 0.001). **(B)** Spearman correlation analysis was used to analyze the correlation between four DNA methyltransferases and MMP14 expression.

MMR is an important correction factor for base mismatches, base deletions, and insertion errors during DNA replication and recombination and plays an important role in maintaining gene stability (29). MMR genes play a crucial role in maintaining the stability of MMR genes. Deletions of some important MMR genes will lead to major errors in DNA replication and recombination, resulting in the occurrence of tumors (30). Therefore, to study the expression of MMP14 and its influence on the cancer process, we chose the MMR of several important genes and the expression level of MMP14 (**Figure 11A**), and we can see from the figure the expression of MMP14 and MMR genes in larger tumors such as KICH, and there were correlations in KIRC, LGG, LIHC, LUSC, OV, PAAD, STAD, TGCT, THCA, and UCEC, of which the most relevant were LGG and UCEC.

A growing number of recent studies have shown that DNA methylation plays a role in the progression of numerous tumors (31, 32). Therefore, in this study, we evaluated the relationship between four DNA methyltransferases and MMP14, as shown in **Figure 11**. The expression of MMP14 is highly correlated with four DNA methyltransferases in many tumors, especially SARC, SKCM, STAD, TGCT, UCEC, BRCA, HNSC, KICH, KIRC, LGG, LIHC, MESO, OV, and PAAD. These data suggest that MMP14 can affect the occurrence and progression of many tumors through DNA MMR and DNA methylation.

### Relationship Between MMP14 Expression and Tumor Mutational Burden and Microsatellite Instability in Endemic Cancer

**Figure 12:**
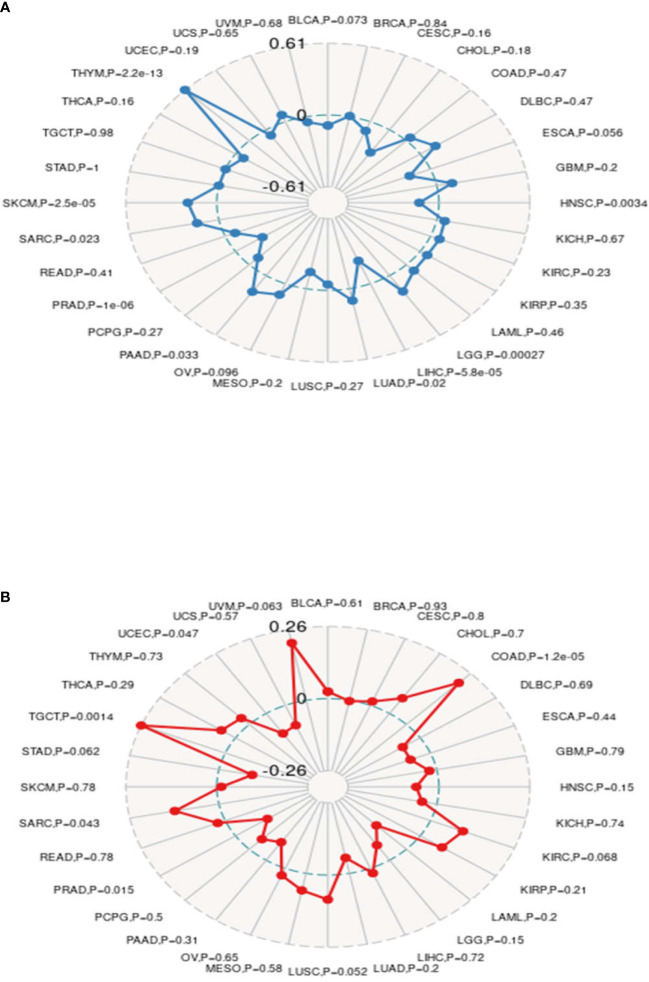
The expression of matrix metalloproteinase 14 (MMP14) was correlated with tumor mutational burden (TMB) and microsatellite instability (MSI). **(A)** Radar images were used to show the correlation between MMP14 expression and TMB. The black value represents the range, and the blue curve represents the correlation coefficient. **(B)** Radar images were used to show the correlation between MMP14 expression and MSI. The black value represents the range, and the red curve represents the correlation coefficient.

Recently, it has been reported that TMB plays an important role as a biomarker in predicting the response to immune checkpoint inhibitors (ICIs) (33–35), such as PD-1/PD-L1. MSI, which also occurs in the vast majority of tumors, may serve as a novel and important biomarker to predict the effects of ICIs (36, 37), as well as PD-1. In this study, we investigated the relationship between MMP14 and TMB and MSI in endemic cancer. The analysis results showed that the expression of MMP14 was positively related to TMB in THYM, SKCM, SARC, PAAD, LUAD, and LGG. In contrast, tumors in which MMP14 expression was negatively correlated with TMB included BLCA, HNSC, LIHC, and PRAD (**Figure 12A**). In terms of MSI, MMP14 expression was positively correlated with MSI in the following tumors: TGCT, SARC, and COAD. The tumors in which MMP14 expression was negatively correlated with MSI included UCEC and PRAD (**Figure 12B**).

## Discussion

The treatment and prevention of cancer are currently a highly significant research direction, and pan-cancer analysis is an important method to compare the differences between different tumors and has great significance and practical value for the discovery of new markers and new effective therapeutic targets of cancer. To date, there have been studies on pan-cancer analysis involving gene mutations, driver genes, gene copy number changes, and tumor purity. These efforts have proven to be of great significance for the treatment and diagnosis of tumors, providing new ideas and perspectives for the treatment and prevention of tumors (29, 38–42).

Numerous studies have shown that the MMP family is involved in the occurrence and progression of a large number of tumors, and MMPs have been shown to be closely related to metastasis and invasion (43). MMP14 is one of the first known MMPs and has been shown to act as an activator of MMP2 (44, 45). Therefore, MMP14 plays an extremely important role in tumors, and strengthening the role of MMP14 across cancers is of great importance and significance for the treatment, prevention, and diagnosis of cancer. Previous studies have shown that high expression of MMP14 is associated with poor prognosis of gastric cancer, colorectal cancer, and liver cancer (44, 46, 47). It has also been suggested that MMP14 can be used as a biological indicator of tumor prognosis (48). Currently, there are still limited studies investigating whether MMP14 is related to tumor prognosis or can be a biomarker for biological prognosis. Whether MMP14 can be used as a biomarker for tumor prognosis needs further study.

In this study, we first analyzed MMP14 expression in generic cancer. We found that compared with normal tissue, MMP14 expression differed in 23 kinds of cancer, and high expression of MMP14 and tumor MMR, MSI, and DNA methylation were closely related to TMB. These factors all play important roles in the progression and prognosis of tumors. At the same time, we found that overexpression of MMP14 was associated with poorer outcomes in a variety of cancers (OS, DSS, PFI, and DFI). In addition, the expression of MMP14 is closely related to the immunoosmosis and ICI level of human pan-cancer, especially in LGG, PRAD, THCA, and other tumors. The results of this study suggest that MMP14 plays a critical role in tumor immunity and may serve as an important biomarker.

There have been some reports on the effect of MMP14 in tumors. Jin et al. (47) indicated that high expression of MMP14 in resectable hepatocellular carcinoma predicted poor prognosis. Dong et al. (44) pointed out that the increased expression of MMP14 is associated with poor prognosis in Chinese gastric cancer patients and that MMP14 plays an important role in the progression and prognosis of gastric cancer and is a convincing biomarker for predicting prognosis in Chinese gastric cancer patients. Cui et al. (46) indicated that MMP14 predicted poor prognosis in patients with colorectal cancer. MMP14 plays an important role in the progression and prognosis of colorectal cancer and may be a useful biomarker for predicting survival after colectomy. At the same time, some studies have shown that MMP14 is involved in normal physiological functions and tumor-related processes such as cell migration, invasion, metastasis, angiogenesis, and proliferation (49, 50). MMP14 has been reported to be upregulated in some cancers (51–53) and to promote invasion and metastasis of cancer cells (52, 54). In this study, the prognosis of MMP14 in pan-cancer patients was analyzed, including OS, DSS, DFI, and PFI. OS analysis of lower MMP14 expression in ACC, BLCA, GBM, HNSC, KIRC, LGG, LIHC, LUAD, MESO, OV, PAAD, and THCA was positively related to the OS prognosis. DSS analysis showed that the low expression of MMP14 was positively correlated with the prognosis of ACC, PAAD, and UCEC. DFI analysis showed that the low expression of MMP14 significantly affected the prognosis of the tumors including ACC, BLCA, BRCA, GBM, KIRC, LGG, LIHC, MESO, OV, PAAD, YHCA, and UVM. PFI analysis of lower MMP14 expression indicated significant effects on the prognosis of BLCA, BRCA, KICH, KIRC, LGG, and LUSC, with the highest correlations observed for ACC, BLCA, BRCA, LGG, PAAD, and KIRC. These data indicate that MMP14 is highly correlated with tumor prognosis. MMP14 is a potential biomarker for the prognosis of pan carcinoma. However, this study did not explore the mechanism of action of MMP14 across cancers, which is a deficiency of this study.

The tumor microenvironment (TME) has recently been widely studied and has become an important consideration in tumor therapy. Numerous studies have confirmed that the TME plays an important role in tumor invasion, micro angiogenesis, tumor proliferation, and even immune escape (55–57). Immune cells are widely present in the matrix of TME cells and play an important role in determining the effect of the TME on tumors. Common and important immune cells include neutrophils, natural killer cells, macrophages, dendritic cells, B cells, and T cells. In the TME, various immune factors are regulated by various key factors while playing their roles (58–60). Moreover, an increasing number of studies have shown that immune cells play important roles in the occurrence and development of many tumors (61–63). However, to date, there have been no studies on the role of MMP14 in the TME. The results of this study showed that a variety of tumors were associated with immune cell infiltration, among which three tumors were most significantly related: BLCA, BRCA, and COAD. This may provide a new direction and target for the treatment and diagnosis of BLCA, BRCA, and COAD. Immune and matrix scores are often used to evaluate the number of infiltrating immune cells in the TME (64). In this study, the expression of MMP14 in BLCA, BRCA, CESC, COAD, DLBC, ESCA, GBM, HNSC, KICH, KIRP, LGG, LIHC, LUAD, LUSC, OV, PAAD, PCPG, PRAD, READ, STAD, and THCA was positively correlated with immune cell score and StromalScore, and the three tumors with the most significant correlation between the expression of MMP14 and immune cell score and StromalScore were BRCA, CESG, and COAD and ImmuneScore were COAD, LGG, and PCPG. In this study, MMP14 was found to be co-expressed with 47 immune checkpoint markers in cancer. MMP14 expression was associated with 36 immune checkpoint genes in LGG, 33 in THCA, and 29 in KICH. We also found that the immune checkpoint gene CD276 was significantly associated with MMP14 expression in 28 of 33 tumors. This evidence suggests that MMP14 can modulate the infiltration of immune cells, perhaps by regulating or recruiting immune cells, and thus play a role in tumor regulation. These results fully prove that MMP14 plays an important role in tumor immunity.

Studies have shown that MMR plays an important role in maintaining the stability and integrity of the whole genome in normal cells. It is generally believed that the main components of MMR are MSH (MutS homologs), and MLH/PMS (MutL homologs) (65). The deletion of the MMR gene will have an important effect on tumor cells, significantly increasing the mutation frequency of related genes in tumor cells. This potentially defective biomarker caused by the deletion of the MMP gene is called MSI (66). A large number of studies have confirmed that MMR gene deletion and MSI are important and sensitive biomarkers for many tumors and play an important role in tumor target prediction and treatment (30, 36, 37). At the same time, a new method for predicting tumor occurrence and progression and DNA methylation and TMB, which includes DNA methylation, involves a mechanism associated with epigenetic change (31, 32). The measurement of cancer cell mutations to predict the prognosis of patients with tumors, especially the ICI response, is of great significance in the treatment of cancer (33–35). In this study, tumors with a high correlation between MMP14 expression and five MMR genes were KICH, KIRC, LGG, LIHC, LUSC, OV, PAAD, STAD, TGCT, THCA, and UCEC, among which LGG and UCEC had the highest correlation. The expression of MMP14 in many tumors, including SARC, SKCM, STAD, TGCT, UCEC, BRCA, HNSC, KICH, KIRC, LGG, LIHC, MESO, OV, and PAAD, is highly correlated with four DNA methyltransferases. At the same time, the expression of MMP14 was positively correlated with TMB in THYM, SKCM, SARC, PAAD, LUAD, and LGG. In contrast, the tumors in which MMP14 expression was negatively correlated with TMB were BLCA, HNSC, LIHC, and PARD. In terms of MSI, MMP14 expression was positively correlated with MSI in the following tumors: TGCT, SARC, and COAD. The tumors in which MMP14 expression was negatively correlated with MSI were UCEC and PARD. All of these data indicate for the first time that MMP14 plays an extremely important role in tumorigenesis and progression and that MMP14 may be an important biomarker for the diagnosis, treatment, and prognosis of multiple tumors.

At present, there have been a few studies on MMP14, but this study is the first to consider MMP14 as a potential target in widespread cancer. At the same time, the results of this study show that MMP14 has an important effect on the prognosis and immune infiltration of many tumors, which provides a new direction for tumor research. At present, the exploration of MMP14 inhibitors continues, but no breakthrough has been made (67). There are many reasons for this. Strengthening studies on the mechanism of MMP14 in tumors should represent a breakthrough in solving this problem, which is also an important deficiency of this paper.

## Conclusion

In conclusion, this study explored the important role of MMP14 in tumor prognosis, and the expression of MMP14 was highly correlated with tumor immune invasion, especially with obvious tumors, including BRCA, CESC, COAD, LGG, and PCPG. Meanwhile, we demonstrate that MMP14 can affect the occurrence and progression of many tumors through TMB, MMR, MSI, and DNA methylation. All of these results fully indicate that MMP14 may be a biomarker for the prognosis, diagnosis, and treatment of many tumors and provide a new idea and direction for subsequent tumor immune research and treatment strategies.

## Data Availability Statement

The original contributions presented in the study are included in the article/**Supplementary Material**. Further inquiries can be directed to the corresponding author.

## Author Contributions

TH conceived and designed the experiments. ML performed the analysis. LY, XW, SL, ZL, SZ, SX organized and collected data. ML and TH wrote the manuscript. TH and BT revised the manuscript. LF and SZ collected references and managed data. All authors contributed to the article and approved the submitted version.

## Funding

This work was supported by the National Natural Science Foundation of China (No. 81460381 and No. 82060246), Key Research and Invention Plan of Jiangxi Science and Technology Department (20192BBG70026), and Natural Science Foundation of Jiangxi Province (S2020ZRMSB1789).

## Conflict of Interest

The authors declare that the research was conducted in the absence of any commercial or financial relationships that could be construed as a potential conflict of interest.

## Publisher’s Note

All claims expressed in this article are solely those of the authors and do not necessarily represent those of their affiliated organizations, or those of the publisher, the editors and the reviewers. Any product that may be evaluated in this article, or claim that may be made by its manufacturer, is not guaranteed or endorsed by the publisher.

## Abbreviations

ACC, adrenocortical carcinoma; BLCA, bladder urothelial carcinoma; BRCA, breast invasive carcinoma; CESC, cervical squamous cell carcinoma and endocervical adenocarcinoma; CHOL, cholangiocarcinoma; COAD, colon adenocarcinoma; DLBC, lymphoid neoplasm diffuse large B-cell lymphoma; ESCA, esophageal carcinoma; GBM, glioblastoma multiforme; HNSC, head and neck squamous cell carcinoma; KICH, kidney chromophobe; KIRC, kidney renal clear cell carcinoma; KIRP, kidney renal papillary cell carcinoma; LAML, acute myeloid leukemia; LGG, brain lower grade glioma; LIHC, liver hepatocellular carcinoma; LUAD, lung adenocarcinoma; LUSC, lung squamous cell carcinoma; MESO, mesothelioma; OV, ovarian serous cystadenocarcinoma; PAAD, pancreatic adenocarcinoma; PCPG, pheochromocytoma and paraganglioma; PRAD, prostate adenocarcinoma; READ, rectum adenocarcinoma; SARC, sarcoma; SKCM, skin cutaneous melanoma; STAD, stomach adenocarcinoma; TGCT, testicular germ cell tumor; THCA, thyroid carcinoma; THYM, thymoma; UCEC, uterine corpus endometrial carcinoma; UCS, uterine carcinosarcoma; UVM, uveal melanoma.

## References

[1] Li M Ren CX Zhang JM Xin XY Hua T Wang HB. The Effects of miR-195-5p/MMP14 on Proliferation and Invasion of Cervical Carcinoma Cells Through TNF Signaling Pathway Based on Bioinformatics Analysis of Microarray Profiling. Cell Physiol Biochem (2018) 50(4):1398–413. doi: 10.1159/000494602 30355924

[2] Pahwa S Stawikowski MJ Fields GB. Monitoring and Inhibiting MT1-MMP During Cancer Initiation and Progression. Cancers (Basel) (2014) 6:416–35. doi: 10.3390/cancers6010416 PMC3980612 24549119

[3] Yadav L Puri N Rastogi V Satpute P Ahmad R Kaur G. Matrix Metalloproteinases and Cancer - Roles in Threat and Therapy. Asian Pac J Cancer Prev (2014) 15:1085–91. doi: 10.7314/APJCP.2014.15.3.1085 24606423

[4] Itoh Y Seiki M. MT1-MMP: A Potent Modifier of Pericellular Microenvironment. J Cell Physiol (2006) 206:1–8. doi: 10.1002/jcp.20431 15920734

[5] Strongin AY. Proteolytic and Non-Proteolytic Roles of Membrane Type-1 Matrix Metalloproteinase in Malignancy. Biochim Biophys Acta (2010) 1803:133–41. doi: 10.1016/j.bbamcr.2009.04.009 PMC2823998 19406172

[6] Lin Y Wang J Jin W Wang L Li H Ma L. NHE1 Mediates Migration and Invasion of HeLa Cells *via* Regulating the Expression and Localization of MT1-MMP. Cell Biochem Funct (2012) 30:41–6. doi: 10.1002/cbf.1815 21997166

[7] Tee YT Liu YF Chang JT Yang SF Chen SC Han CP. Single-Nucleotide Polymorphisms and Haplotypes of Membrane Type 1-Matrix Metalloproteinase in Susceptibility and Clinical Significance of Squamous Cell Neoplasia of Uterine Cervix in Taiwan Women. Reprod Sci (2012) 19:932–8. doi: 10.1177/1933719112438445 22527983

[8] Liu T Zhang X Gao S Jing F Yang Y Du L. Exosomal Long Noncoding RNA CRNDE-H as a Novel Serum-Based Biomarker for Diagnosis and Prognosis of Colorectal Cancer. Oncotarget (2016) 7:85551–63. doi: 10.18632/oncotarget.13465 PMC5356757 27888803

[9] Wang H Zhang X Huang L Li J Qu S Pan F. Matrix Metalloproteinase-14 Expression and Its Prognostic Value in Cervical Carcinoma. Cell Biochem Biophys (2014) 70:729–34. doi: 10.1007/s12013-014-9974-8 24789545

[10] Vos MC van der Wurff AAM van Kuppevelt TH Massuger LFAG. The Role of MMP-14 in Ovarian Cancer: A Systematic Review. J Ovarian Res (2021) 14(1):101. doi: 10.1186/s13048-021-00852-7 34344453 PMC8336022

[11] Ogawa S Kubo H Murayama Y Kubota T Yubakami M Matsumoto T. Matrix Metalloprotease-14 Is a Target Enzyme for Detecting Peritoneal Metastasis in Gastric Cancer. Photodiagnosis Photodyn Ther (2021) 35:102420. doi: 10.1016/j.pdpdt.2021.102420 34242818

[12] Ozaki S Umakoshi A Yano H Ohsumi S Sumida Y Hayase E. Chloride Intracellular Channel Protein 2 Is Secreted and Inhibits MMP14 Activity, While Preventing Tumor Cell Invasion and Metastasis. Neoplasia (2021) 23(8):754–65. doi: 10.1016/j.neo.2021.06.001 PMC8260957 34229297

[13] Sun W Zhang Y Liu B Duan Y Li W Chen J. Gene Polymorphism of MUC15, MMP14, BRAF, and COL1A1 Is Associated With Capsule Formation in Hepatocellular Carcinoma. Can J Gastroenterol Hepatol (2021) 2021:9990305. doi: 10.1155/2021/9990305 34007838 PMC8100414

[14] Altadill A Eiro N González LO Andicoechea A Fernández-Francos S Rodrigo L. Relationship Between Metalloprotease-7 and -14 and Tissue Inhibitor of Metalloprotease 1 Expression by Mucosal Stromal Cells and Colorectal Cancer Development in Inflammatory Bowel Disease. Biomedicines (2021) 9(5):495. doi: 10.3390/biomedicines9050495 33946534 PMC8147221

[15] Pach E Brinckmann J Rübsam M Kümper M Mauch C Zigrino P. Fibroblast MMP14-Dependent Collagen Processing Is Necessary for Melanoma Growth. Cancers (Basel) (2021) 13(8):1984. doi: 10.3390/cancers13081984 33924099 PMC8074311

[16] Bindea G Mlecnik B Tosolini M Kirilovsky A Waldner M Obenauf AC. Spatiotemporal Dynamics of Intratumoral Immune Cells Reveal the Immune Landscape in Human Cancer. Immunity (2013) 39:782–95. doi: 10.1016/j.immuni.2013.10.003 24138885

[17] Finn OJ. Cancer Immunology. N Engl J Med (2008) 358:2704–15. doi: 10.1056/NEJMra072739 18565863

[18] Gajewski TF Schreiber H Fu YX. Innate and Adaptive Immune Cells in the Tumor Microenvironment. Nat Immunol (2013) 14:1014–22. doi: 10.1038/ni.2703 PMC4118725 24048123

[19] Topalian SL Drake CG Pardoll DM. Immune Checkpoint Blockade: A Common Denominator Approach to Cancer Therapy. Cancer Cell (2015) 27:450–61. doi: 10.1016/j.ccell.2015.03.001 PMC4400238 25858804

[20] Quail DF Joyce JA. Microenvironmental Regulation of Tumor Progression and Metastasis. Nat Med (2013) 19:1423–37. doi: 10.1038/nm.3394 PMC3954707 24202395

[21] Lee KH Kim EY Yun JS Park YL Do SI. The Prognostic and Predictive Value of Tumor Infiltrating Lymphocytes and Hematologic Parameters in Patients With Breast Cancer. BMC Cancer (2018) 18:938. doi: 10.1186/s12885-018-4832-5 30285668 PMC6167816

[22] Lee N Zakka LR Mihm MC Jr. Schatton T. Tumour Infiltrating Lymphocytes in Melanoma Prognosis and Cancer Immunotherapy. Pathology (2016) 48:177–87. doi: 10.1016/j.pathol.2015.12.006 27020390

[23] Gordon SR Maute RL Dulken BW Hutter G George BM McCracken MN. PD-1 Expression by Tumour-Associated Macrophages Inhibits Phagocytosis and Tumour Immunity. Nature (2017) 545:495–9. doi: 10.1038/nature22396 PMC5931375 28514441

[24] Mizuno H Kitada K Nakai K Sarai A. PrognoScan: A New Database for Meta-Analysis of the Prognostic Value of Genes. BMC Med Genomics (2009) 2:18. doi: 10.1186/1755-8794-2-18 19393097 PMC2689870

[25] Tang Z Li C Kang B Gao G Li C Zhang Z. GEPIA: A Web Server for Cancer and Normal Gene Expression Profiling and Interactive Analyses. Nucleic Acids Res (2017) 45:W98–W102. doi: 10.1093/nar/gkx247 28407145 PMC5570223

[26] Hou GX Liu P Yang J Wen S. Mining Expression and Prognosis of Topoisomerase Isoforms in Non-Small-Cell Lung Cancer by Using Oncomine and Kaplan-Meier Plotter. PloS One (2017) 12:e0174515. doi: 10.1371/journal.pone.0174515 28355294 PMC5371362

[27] Li T Fan J Wang B Traugh N Chen Q Liu JS. TIMER: A Web Server for Comprehensive Analysis of Tumor-Infiltrating Immune Cells. Cancer Res (2017) 77(21):e108–10. doi: 10.1158/0008-5472.CAN-17-0307 PMC6042652 29092952

[28] Li B Severson E Pignon J-C Zhao H Li T Novak J. Comprehensive Analyses of Tumor Immunity: Implications for Cancer Immunotherapy. Genome Biol (2016) 17(1):174. doi: 10.1186/s13059-016-1028-7 27549193 PMC4993001

[29] Li GM. Mechanisms and Functions of DNA Mismatch Repair. Cell Res (2008) 18:85–98. doi: 10.1038/cr.2007.115 18157157

[30] Baretti M Le DT. DNA Mismatch Repair in Cancer. Pharmacol Ther (2018) 189:45–62. doi: 10.1016/j.pharmthera.2018.04.004 29669262

[31] Klutstein M Nejman D Greenfield R Cedar H. DNA Methylation in Cancer and Aging. Cancer Res (2016) 76:3446–50. doi: 10.1158/0008-5472.Can-15-3278 27256564

[32] Koch A Joosten SC Feng Z de Ruijter TC Draht MX Melotte V. Analysis of DNA Methylation in Cancer: Location Revisited. Nat Rev Clin Oncol (2018) 15:459–66. doi: 10.1038/s41571-018-0004-4 29666440

[33] Yarchoan M Hopkins A Jaffee EM. Tumor Mutational Burden and Response Rate to PD-1 Inhibition. N Engl J Med (2017) 377:2500–1. doi: 10.1056/NEJMc1713444 PMC6549688 29262275

[34] Chan TA Yarchoan M Jaffee E Swanton C Quezada SA Stenzinger A. Development of Tumor Mutation Burden as an Immunotherapy Biomarker: Utility for the Oncology Clinic. Ann Oncol (2019) 30:44–56. doi: 10.1093/annonc/mdy495 30395155 PMC6336005

[35] Samstein RM Lee CH Shoushtari AN Hellmann MD Shen R Janjigian YY. Tumor Mutational Load Predicts Survival After Immunotherapy Across Multiple Cancer Types. Nat Genet (2019) 51:202–6. doi: 10.1038/s41588-018-0312-8 PMC6365097 30643254

[36] Dudley JC Lin MT Le DT Eshleman JR. Microsatellite Instability as a Biomarker for PD-1 Blockade. Clin Cancer Res (2016) 22:813–20. doi: 10.1158/1078-0432.Ccr-15-1678 26880610

[37] Hause RJ Pritchard CC Shendure J Salipante SJ. Classification and Characterization of Microsatellite Instability Across 18 Cancer Types. Nat Med (2016) 22:1342–50. doi: 10.1038/nm.4191 27694933

[38] Zack TI Schumacher SE Carter SL Cherniack AD Saksena G Tabak B. Pan-Cancer Patterns of Somatic Copy Number Alteration. Nat Genet (2013) 45:1134–40. doi: 10.1038/ng.2760 PMC3966983 24071852

[39] Aran D Sirota M Butte AJ. Systematic Pan-Cancer Analysis of Tumour Purity. Nat Commun (2015) 6:8971. doi: 10.1038/ncomms9971 26634437 PMC4671203

[40] Ma X Liu Y Liu Y Alexandrov LB Edmonson MN Gawad C. Pan-Cancer Genome and Transcriptome Analyses of 1,699 Paediatricleukaemias and Solid Tumours. Nature (2018) 555:371–6. doi: 10.1038/nature25795 PMC5854542 29489755

[41] Saghafinia S Mina M Riggi N Hanahan D Ciriello G. PanCancer Landscape of Aberrant DNA Methylation Across Human Tumors. Cell Rep (2018) 25:1066–80.e8. doi: 10.1016/j.celrep.2018.09.082 30355485

[42] Priestley P Baber J Lolkema MP Steeghs N de Bruijn E Shale C. Pan-Cancer Whole-Genome Analyses of Metastatic Solid Tumours. Nature (2019) 575:210–6. doi: 10.1038/s41586-019-1689-y PMC6872491 31645765

[43] Sato H Takino T Okada Y Cao J Shinagawa A Yamamoto E. A Matrix Metalloproteinase Expressed on the Surface of Invasive Tumour Cells. Nature (1994) 370:61–5. doi: 10.1038/370061a0 8015608

[44] Dong Y Chen G Gao M Tian X. Increased Expression of MMP14 Correlates With the Poor Prognosis of Chinese Patients With Gastric Cancer. Gene (2015) 563(1):29–34. doi: 10.1016/j.gene.2015.03.003 25748728

[45] Hui P Xu X Xu L Hui G Wu S Lan Q. Expression of MMP14 in Invasive Pituitary Adenomas: Relationship to Invasion and Angiogenesis. Int J Clin Exp Pathol (2015) 8:3556–7. PMC4466925 26097538

[46] Cui G Cai F Ding Z Gao L. MMP14 Predicts a Poor Prognosis in Patients With Colorectal Cancer. Hum Pathol (2019) 83:36–42. doi: 10.1016/j.humpath.2018.03.030 30120968

[47] Jin Y Liang ZY Zhou WX Zhou L. High MMP14 Expression Is Predictive of Poor Prognosis in Resectable Hepatocellular Carcinoma. Pathology (2020) 52(3):359–65. doi: 10.1016/j.pathol.2020.01.436 32122646

[48] Zhang H Liu M Sun Y Lu J. MMP-14 Can Serve as a Prognostic Marker in Patients With Supraglottic Cancer. Eur Arch Otorhinolaryngol (2009) 266(9):1427–34. doi: 10.1007/s00405-009-0943-6 19283401

[49] Merchant N Nagaraju GP Rajitha B Lammata S Jella KK Buchwald ZS. Matrixmetalloproteinases:their Functional Role in Lung Cancer. Carcinogenesis (2017) 38(8):766–80. doi: 10.1093/carcin/bgx063 28637319

[50] Utispan K Niyomtham N Yingyongnarongkul BE Koontongkaew S. Ethanolic Extract of Ocimum Sanctum Leaves Reduced Invasion and Matrix Metalloproteinase Activity of Head and Neck Cancer Cell Lines. Asian Pac J Cancer Prev (2020) 21(2):363–70. doi: 10.31557/APJCP.2020.21.2.363 PMC7332114 32102512

[51] Hu Y Wu F Liu Y Zhao Q Tang H. DNMT1 Recruited by EZH2-Mediated Silencing of miR-484 Contributes to the Malignancy of Cervical Cancer Cells Through MMP14 and HNF1A. Clin Epigenet (2019) 11(1):186. doi: 10.1186/s13148-019-0786-y PMC6898970 31810492

[52] Kudelski J Młynarczyk G Darewicz B Bruczko-Goralewska M Romanowicz L. Dominative Role of MMP-14 Over MMP-15 in Human Urinary Bladder Carcinoma on the Basis of Its Enhanced Specific Activity. Med (Baltimore) (2020) 99(7):e19224. doi: 10.1097/MD.0000000000019224 PMC7035044 32049862

[53] Zhang Q Lou L Cai X Hao Z Nie S Liu Y. Clinical Significance of AJUBA, YAP1, and MMP14 Expression in Esophageal Squamous Cell Carcinoma. Int J Clin Exp Pathol (2018) 11(12):6018–24. PMC6963081 31949690

[54] Moch H Cubilla AL Humphrey PA Reuter VE Ulbright TM. The 2016 WHO Classification of Tumours of the Urinary System and Male Genital Organs-Part A:Renal,Penile,and Testicular Tumours. Eur Urol (2016) 70(1):93–105. doi: 10.1016/j.eururo.2016.02.029 26935559

[55] Altorki NK Markowitz GJ Gao D Port JL Saxena A Stiles B. The Lung Microenvironment: An Important Regulator of Tumour Growth and Metastasis. Nat Rev Cancer (2019) 19:9–31. doi: 10.1038/s41568-018-0081-9 30532012 PMC6749995

[56] Sautes-Fridman C Petitprez F Calderaro J Fridman WH. Tertiary Lymphoid Structures in the Era of Cancer Immunotherapy. Nat Rev Cancer (2019) 19:307–25. doi: 10.1038/s41568-019-0144-6 31092904

[57] Fane M Weeraratna AT. How the Ageing Microenvironment Influences Tumour Progression. Nat Rev Cancer (2020) 20:89–106. doi: 10.1038/s41568-019-0222-9 31836838 PMC7377404

[58] Xu SJ Hu HT Li HL Chang S. The Role of miRNAs in Immune Cell Development, Immune Cell Activation, and Tumor Immunity: With a Focus on Macrophages and Natural Killer Cells. Cells (2019) 8:1140. doi: 10.3390/cells8101140 PMC6829453 31554344

[59] Chen Y Meng Z Zhang L Liu F. CD2 Is a Novel Immune-Related Prognostic Biomarker of Invasive Breast Carcinoma That Modulates the Tumor Microenvironment. Front Immunol (2021) 12:664845. doi: 10.3389/fimmu.2021.664845 33968066 PMC8102873

[60] Zhou B Gao S. Pan-Cancer Analysis of FURIN as a Potential Prognostic and Immunological Biomarker. Front Mol Biosci (2021) 8:648402. doi: 10.3389/fmolb.2021.648402 33968987 PMC8100462

[61] Rabinovich GA Gabrilovich D Sotomayor EM. Immunosuppressive Strategies That Are Mediated by Tumor Cells. Annu Rev Immunol (2007) 25:267–96. doi: 10.1146/annurev.immunol.25.022106.141609 PMC2895922 17134371

[62] Beatty GL Gladney WL. Immune Escape Mechanisms as a Guide for Cancer Immunotherapy. Clin Cancer Res (2015) 21:687–92. doi: 10.1158/1078-0432.Ccr-14-1860 PMC4334715 25501578

[63] Osipov A Saung MT Zheng L Murphy AG. Small Molecule Immunomodulation: The Tumor Microenvironment and Overcoming Immune Escape. J Immunother Cancer (2019) 7:224. doi: 10.1186/s40425-019-0667-0 31439034 PMC6704558

[64] Galon J Angell HK Bedognetti D Marincola FM. The Continuum of Cancer Immunosurveillance: Prognostic, Predictive, and Mechanistic Signatures. Immunity (2013) 39:11–26. doi: 10.1016/j.immuni.2013.07.008 23890060

[65] Fishel R. Mismatch Repair. J Biol Chem (2015) 290:26395–403. doi: 10.1074/jbc.R115.660142 PMC4646297 26354434

[66] Russo M Crisafulli G Sogari A Reilly NM Arena S Lamba S. Adaptive Mutability of Colorectal Cancers in Response to Targeted Therapies. Science (2019) 366:1473–80. doi: 10.1126/science.aav4474 31699882

[67] Ling B Watt K Banerjee S Newsted D Truesdell P Adams J. A Novel Immunotherapy Targeting MMP-14 Limits Hypoxia, Immune Suppression and Metastasis in Triple-Negative Breast Cancer Models. Oncotarget (2017) 8:58372–85. doi: 10.18632/oncotarget.17702 PMC5601659 28938563

