# Supplementary figures and images for: Immune Infiltration of MMP14 in Pan Cancer and Its Prognostic Effect on Tumors
Source: Front Oncol. 2021 Sep 17;11:717606. doi: 10.3389/fonc.2021.717606 (PMC8484967; doi:10.3389/fonc.2021.717606)

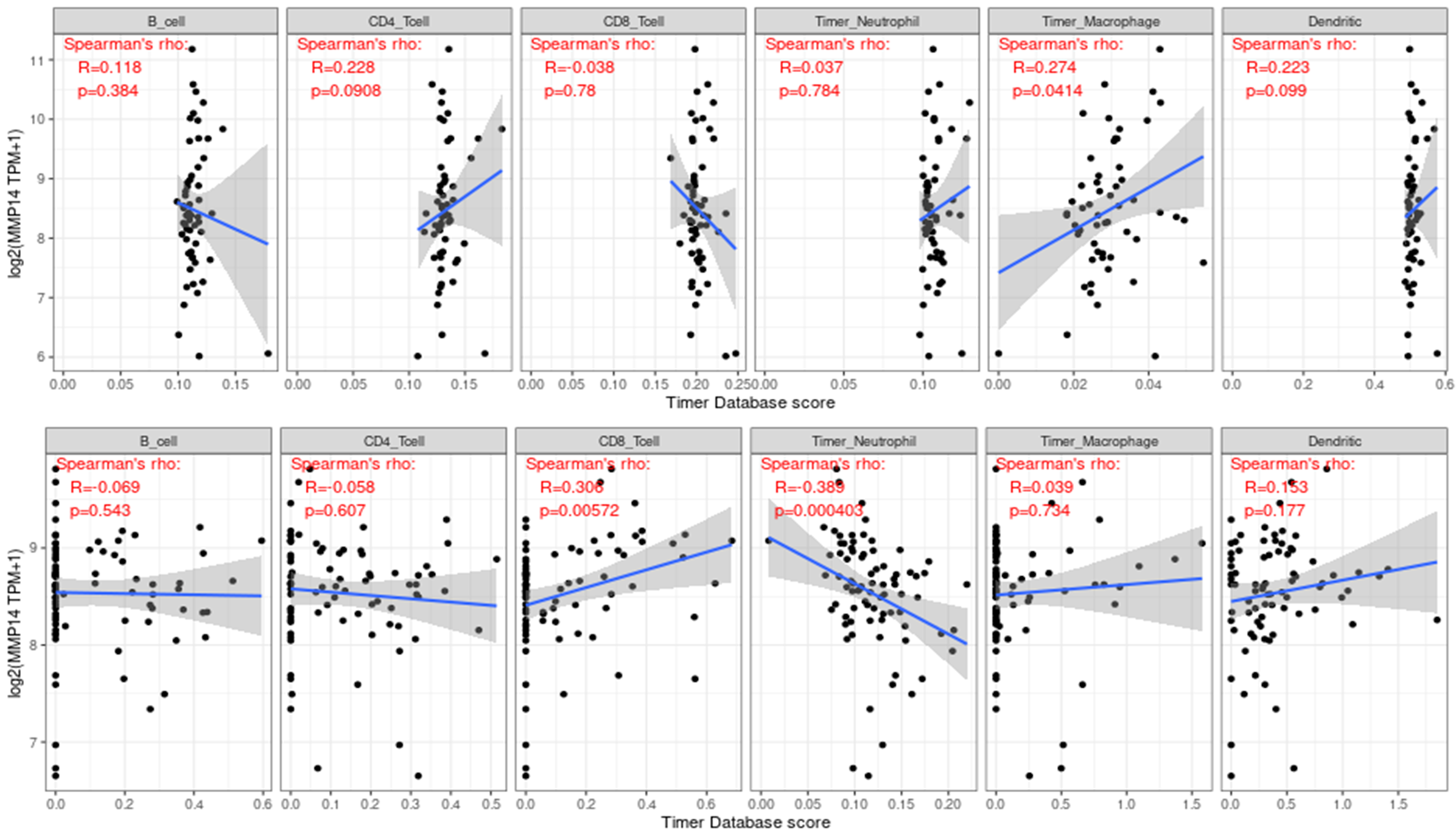

Supplement: Supplementary file 1 [file Image_1.tif]

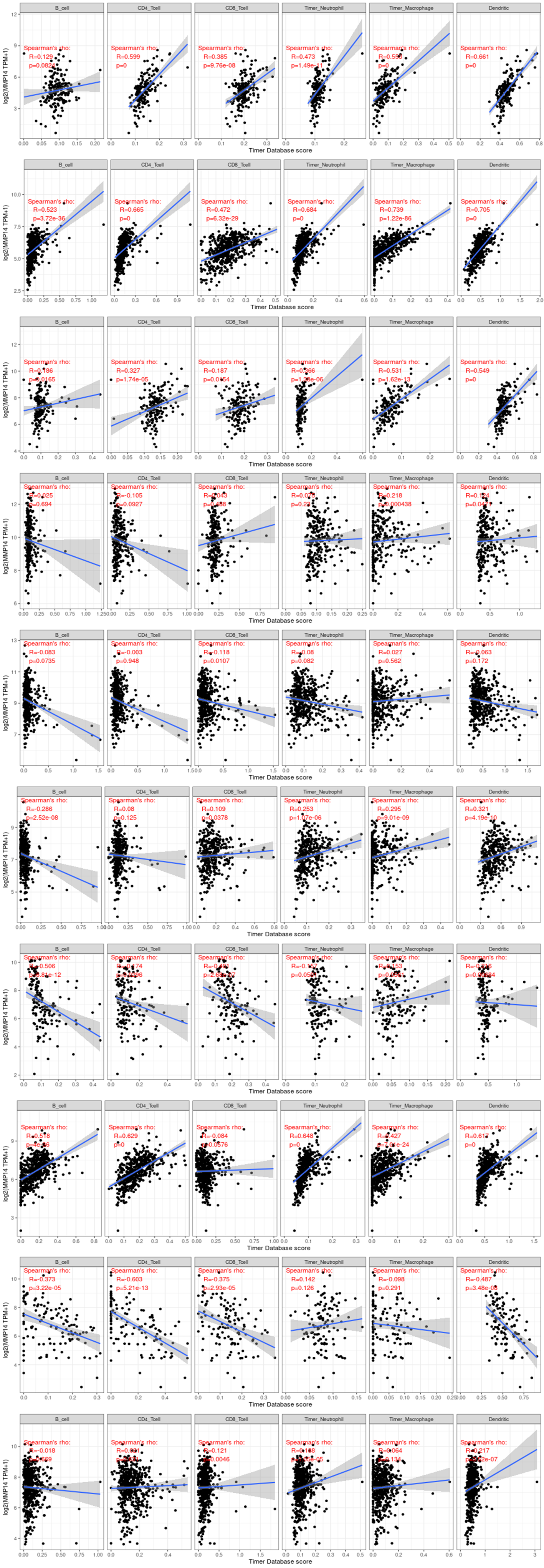

Supplement: Supplementary file 3 [file Image_3.tif]
